# Sodium nitrate has no detrimental effect on milk fatty acid profile and rumen bacterial population in water buffaloes

**DOI:** 10.1186/s13568-022-01350-9

**Published:** 2022-02-05

**Authors:** Fang Xie, Zhenhua Tang, Xin Liang, Chongli Wen, Mengwei Li, Yanxia Guo, Kaiping Peng, Chengjian Yang

**Affiliations:** Key Laboratory of Buffalo Genetics, Breeding and Reproduction Technology, Ministry of Agriculture and Guangxi, Buffalo Research Institute, Chinese Academy of Agricultural Sciences, 24-1Yongwu Road, Nanning, 530001 People’s Republic of China

**Keywords:** Sodium nitrate, Fatty acids profile, Methane, Rumen, Water buffalo

## Abstract

This study evaluated the influence of dietary sodium nitrate on ruminal fermentation profiles, milk production and composition, microbial populations and diversity in water buffaloes. Twenty-four female water buffaloes were randomly divided into four groups and fed with 0, 0.11, 0.22, 044 g sodium nitrate per kg body weight diets, respectively. Results showed that the concentration of acetate, propionate, butyrate and total VFA in all sodium nitrate–adapted water buffaloes were greater than the control group (*P*  < 0.05). Although the milk fatty acids value at 0.11 g sodium nitrate/kg/d were slightly lower than other treatments, no significant differences were observed among different treatments (*P * > 0.05). Compared to the control group, the archaea richness (ace and chao1) and diversity (Shannon index) indices were increased by nitrate supplementation (*P*  < 0.05). Compared with the control group, sodium nitrate did not affect bacterial abundance at the phylum and genus level, but the relative abundance of the methanogen genera was greatly changed. There was a tendency for *Methanobrevibacter* to decrease in the sodium nitrate group (*P*  = 0.091). Comparisons of archaea communities by PCoA analysis showed significant separation between the control group and nitrate treatments (*P * = 0.025). It was concluded that added 0.11–0.44 g sodium nitrate/kg of body weight increased the rumen VFA production and archaeal diversity of water buffaloes but had no detrimental effect on milk yield or composition, fatty acids profile, rumen methanogen or *Butyrivibrio* group population related to biohydrogenation.

## Introduction

It is reported that approximate 14.5% of all anthropogenic emissions were emitted by the livestock sector every year and has a significant negative effect on climate change (Gerber et al. 2013). Methane is the second-largest greenhouse gas and representing 16% of total global greenhouse gas emissions (Pachauri et al. 2014). Methane emission from enteric fermentation from ruminants is a dominant source of greenhouse gas. In the rumen, methanogenic archaea responsible for methanogenesis uses carbon dioxide and hydrogen as main substrates. Methane reduction from ruminants by different strategies has been investigated in many studies (Buddle et al. 2011; Zhou et al. 2011; Guyader et al. 2015; Klop et al. 2016). The efficiency of nitrate supplementation on methane reduction in ruminants has been confirmed both in vivo and in vitro (Nolan et al. 2010; Lee and Beauchemin 2014; Yang et al. 2016). However, the effects of inhibition of methanogenesis on animal product quality still need to be evaluated.

Buffalo’s milk is the second-largest milk type of the world after cow milk (FAO 2020) and has higher fat, protein, minerals and conjugated linoleic acid (CLA) contents than cow milk (Ahmad et al. 2013). In ruminant meat and milk, saturated fatty acids were produced from the biohydrogenation of unsaturated fatty acids and were harmful to human health (Givens and Shingfield 2004). Both molecular and metabolic hydrogen can be utilized by ruminal microorganisms during methanogenesis and the biohydrogenation of unsaturated fatty acids. Many researches have been undertaken to find sustainable ways of lowering methane emissions from ruminants. The effect of dietary nitrate on methane reduction appears effective and consistent in both in vitro and in vivo studies and also persistent in several long-term studies. Nitrates on dairy farms may come from the groundwater and plants which fertilized by commercial fertilizer and/or animal wastes. It may be consumed by grazing ruminants on a daily basis. We found sodium nitrate may lead to lower butyrate production and increased CLA passage of the rumen in vitro, however, overall biohydrogenation was not affected by methanogenesis inhibition (Yang et al. 2019). The interactions between methanogenesis and biohydrogenation in vivo are still unclear. As far as we know, the consequences of methanogenesis were inhibited by sodium nitrate on biohydrogenation in water buffaloes are still unclear. Therefore, here we assess the influence of dietary sodium nitrate supplementation on ruminal fermentation profiles, milk production and composition, microbial populations and diversity in water buffaloes.

## Materials and methods

This study was conducted at the research farm of Buffalo Research Institute, Chinese Academy of Agricultural Sciences. All animal procedures were performed according to the BRI-CAAS (Buffalo Research Institute, Chinese Academy of Agricultural Sciences) guidelines on animal experiments.

### Animals and experimental design

Twenty-four healthy lactating Murrah water buffaloes with the initial body weight of 614 ± 26 kg was used for this study. Water buffaloes were randomly allocated to four experimental treatments. There are six replicates per treatment and one water buffalo per replicate. The four diets are as follows: basal diet without sodium nitrate (control group), basal diet with 0.11 g sodium nitrate/kg of body weight (low nitrate diet), basal diet with 0.22 g sodium nitrate/kg of body weight (medium nitrate diet), basal diet with 0.44 g sodium nitrate/kg of body weight (high nitrate diet). The purity of sodium nitrate was 99% (Yuan Feng Co., Ltd., Zhengzhou City, China). The dosages of sodium nitrate were based on the results of the previous studies with dairy cows and beef cattle (Lee and Beauchemin 2014). Urea was used to maintain an isonitrogenous intake of treatments. The sodium nitrate and urea were mixed with diets before being fed to animals. The nutritional levels of the basal diets are presented in Table 1. All animals were housed in individual stalls for 56 days which included a 28-days adaptation period. In order to avoid the risk of intoxication, animals receiving diets containing sodium nitrate were acclimatized gradually to the adaptation period. The diets were offered twice daily at 0600 h and 1400 h. Milk, ruminal contents and blood samples were collected during the following 28 days. All water buffaloes had free access to freshwater during the entire experimental period.

**Table 1:** Constituents and nutrient concentrations in basal diet for water buffaloes

Items	DM, %
Cassava residues	13.00
Elephant grass	10.00
Brewer’s grain	10.00
Corn silage	17.00
Corn	26.75
Soybean meal	3.00
Wheat bran	7.50
Cottonseed meal	8.50
CaHPO_4_	0.75
Shell meal	0.50
NaCl	1.00
NaHCO_3_	1.50
Premix	0.50
Total	100
Nutrient concentration	
GE MJ/kg	16.14
CP%	11.74
NDF%	29.46
ADF%	16.77

Nutrient concentrations were measured according the methods described in reference (Zhang 2007); each kg premix contained: vitamin E 3000 IU, vitamin D 150,000 IU, vitamin A 500,000 IU, Cu 1.3 g, Fe 4.0 g, Mn 3.0 g, I 80 mg, Zn 6.0 g, Co 80 mg, Se 50 mg

### Sample collection

Feed intake was measured and representative feed samples were taken for further chemical analysis during the last week of the measurements period. Water buffaloes were milked twice daily (0600 and 1600 h). Milk yields were recorded every day. Approximately 500 ml of milk samples were taken from each water buffalo each week for milk composition and fatty acids analysis. Three water buffaloes from each group were randomly selected and used as rumen content donors on the last day of the experimental duration. Approximately 250 ml ruminal contents were collected before morning feeding with stomach tubing. Ruminal contents were immediately transferred to the laboratory with an icebox. During the experimental period, blood samples were collected from each water buffalo via the jugular vein in heparinized collection tubes before the morning feeding. Haemoglobin and methaemoglobin content in blood samples were analyzed within 2 h after sampling using MetHb and Hb kits (Gu Duo Sheng Wu, Shanghai).

Five ml of ruminal contents were kept at – 20 ℃ for later DNA extraction and further microbial population and microbial diversity analysis. The remaining ruminal contents were squeezed through two layers of cheesecloth; One 10 ml filtrate was acidified with 1 ml 0.5 mol/l HCl and kept at – 20 ℃ for subsequent ruminal NH_3_–N analysis. One 5 ml filtrate was kept at – 20 ℃ for subsequent microbial crude protein analysis. Two ml of freshly prepared 25% meta-phosphoric acid were added to 8 ml of filtrate, after centrifuged (12,000 rpm, 10 min), the supernatant fluid was used for volatile fatty acids determination.

### Feed, milk and rumen fermentation samples composition analysis

Ruminal pH was determined immediately after samples collection using a portable pH meter (HI 9024C, Hanna Instruments, USA). Milk composition was determined by MilkoScan F120 (FOSS, Denmark). Milk fatty acids were quantified by gas chromatography (Yang et al. 2019). VFA concentration was also measured by gas chromatography (Qin 1982). Microbial crude protein (MCP) also measured by the method of Makkar et al. (1982). NH_3_–N measured by the phenol-hypochlorite method (Weatherburn 1967). Microbial DNA was extracted from rumen contents by the bead-beating method (Yu and Morrison 2004). DNA concentration and purity were determined using a NanoDrop 1000 UV–vis spectrophotometer (Thermo Scientific, USA).

### Primers and real-time PCR of microbial population

The PCR primers for total bacteria, methanogens, protozoa, fungi, *B. proteoclasticus*, *B. fibrsolvens*  +  *Pseudobutyrivibrio* spp., ‘*Atypical*’ *Butyrivibrio* and *B. hungatei* were as listed in Table 2 (Sylvester et al. 2004; Denman and McSweeney 2006; Denman et al. 2007; Shingfield et al. 2012). Amplification condition was as follows: initial denaturation at 95 ℃ for 3 min, followed by 40 cycles of denaturation at 95 ℃for 15 s, annealing at the respective annealing temperature for 60 s, except for the *B. proteoclasticus* and the *B. hungatei* primer sets, where the following protocol was used: initial denaturation at 95 ℃ for 3 min, then 40 cycles at 95 ℃ for 15 s, annealing at the respective annealing temperature for 30 s, and extension at 72 ℃ for 30 s (Shingfield et al. 2012). PCR products concentration were measured with a NanoDrop 1000 UV–vis spectrophotometer (Thermo Scientific, USA). For each standard, copy number concentration was calculated based on the length of the PCR product and the mass concentration (Yu et al. 2005). The real-time PCR was carried out using a Roche-480 II system with fluorescence detection of SYBR green dye. The target DNA was quantified using serial ten-fold dilutions from 10^–1^ to 10^–10^ DNA copies of the previously quantified DNA standards.

**Table 2:** PCR primers for real time PCR

Target species	Forward/reverse	Primer sequences (5′– > 3′)	References
Total bacteria	F	*CGGCAACGAGCGCAACCC*	Denman and McSweeney 2006
	R	*CCATTGTAGCACGTGTGTAGCC*	
Methanogens	F	*TTCGGTGGATCDCARAGRGC*	Denman et al. 2007
	R	*GBARGTCGWAWCCGTAGAATCC*	
Protozoal	F	*GCTTTCGWTGGATGTGTATT*	Sylvester et al. 2004
	R	*CTTGCCCTCYAATCGTWCT*	
Fungi	F	*GAGGAAGTAAAAGTCGTAACAAGGTTTC*	Denman and McSweeney 2006
	R	*CAAATTCACAAAGGGTAGGATGATT*	
*B. proteoclasticus*	*CprF*	*TCCGGTGGTATGAGATGGGC*	Shingfield et al. 2012
	*CprR*	*GTCGCTGCATCAGAGTTTCCT*	
	*CprP*	*CCGCTTGGCCGTCCGACCTCTCAGTCCGAGCGG*	
*B. fibrisolvens* + *Pseudobutyrivibrio* spp.	*BfiF*	*GCCTCAGCGTCAGTAATCG*	Shingfield et al. 2012
	*BfiR*	*GGAGCGTAGGCGGTTTTAC*	
‘*Atypical*’* Butyrivibrio*	*AtbF*	*GACGGTGTATCAAGTCTGAAGTG*	Shingfield et al. 2012
	*AtbR*	*GCCGGCACTGAAAGACTATGTC*	
*B. hungatei*	*BhuF*	*AGGGTAATGCCTGTAGCTC*	Shingfield et al. 2012
	*BhuR*	*TCACCCTCGCGGGAT*	

### 16S RNA gene amplification

The variable region V3-V4 of the bacterial 16S rRNA gene was amplified with primers 338F (5′-ACTCCTACGGGAGGCAGCAG-3′) and 806R (5′-GGACTACHVGGGTWTCTAAT-3′). The variable V4 region of the archaeal 16S rRNA gene was amplified using the primers Arch349F (5′-GYGCASCAGKCGMGAAW-3′) and Arch806R (5′-GGACTACVSGGGTATCTAAT-3′) (Takai and Horikoshi 2000). PCR amplification of 16S rRNA genes was performed as follows: initial denaturation at 95 ℃ for 3 min, followed by 27 cycles of denaturing at 95 ℃ for 30 s, annealing at 55 ℃ for 30 s and extension at 72 ℃ for 45 s, and single extension at 72 ℃ for 10 min. PCR reactions were performed in triplicate. The PCR product was extracted from 2% agarose gel and purified using the AxyPrep DNA Gel Extraction Kit (Axygen Biosciences, USA) according to manufacturer’s instructions and quantified using a Quantus™ Fluorometer (Promega, USA).

### Illumina MiSeq sequencing

Purified amplicons were pooled in equimolar proportions and paired-end sequenced (2 × 300) on an Illumina MiSeq platform (Illumina, USA) according to the standard protocols by Majorbio Bio-Pharm Technology Co. Ltd. (Shanghai, China).

### Processing of sequencing data

The raw 16S rRNA gene sequencing reads were demultiplexed, quality-filtered by Trimmomatic and merged by FLASH. Operational taxonomic units (OTUs) with 97% similarity cutoff were clustered using UPARSE (version 7.1, http://drive5.com/uparse/), and chimeric sequences were identified and removed. The taxonomy of each OTU representative sequence was analyzed by RDP Classifier (http://rdp.cme.msu.edu/) against the SILVA 16S rRNA database (Release123) using the confidence threshold of 0.7. The analysis was performed using the free online platform, Majorbio I-sanger Cloud Platform (https:// www.i-sanger.com).

### Statistical analysis

The statistical analysis of alpha and beta diversity of ruminal bacterial community composition was carried out in Mothur (version v.1.30.1) (Schloss et al. 2011). The influence of sodium nitrate on growth performance, rumen fermentation parameters, microbial population and composition were analyzed using a one-way ANOVA procedure in SPSS 16.0. Data were reported as least-squared means and standard error of means (SEM). Differences between treatments were tested using Tukey’s multiple comparison tests. Differences were considered significant at *P*  < 0.05. Correlation analysis among the milk yield, milk composition, pH, VFA, NH_3_–N, MCP concentration and microbial proportions was performed using Spearman’s rank correlation, and significant differences were declared at *P*  < 0.05.

## Results

### DMI, blood methaemoglobin, milk yield and milk composition

As shown in Table 3, compared to the control group (0 g nitrate/kg body weight), the nitrate additive treatments did not influence dry matter intake (DMI), milk yield and protein (*P*  > 0.05). Methaemoglobin percentages of this experiment ranged from 10.2 to 17.8%, with no significant difference among treatments (*P*  > 0.05). Although the effects of nitrate on milk fat and total solid contents were not significant between control and nitrate treatments (*P*  > 0.05), compared to the control group, sodium nitrate at 0.11 g nitrate/kg body weight caused a decrease in milk fat and total solid contents and an increase on lactose, while nitrate at 0.22 or 0.44 g nitrate/kg body weight caused an increase on fat and total solid content.

**Table 3:** Effects of different inclusion rate of sodium nitrate on dry matter intake, milk yield and milk composition in water buffaloes

Item	Sodium nitrate, g/kg body weight				SE	*P*
	0	0.11	0.22	0.44		
DMI (kg/day)	13.66	13.08	13.06	14.08	0.24	0.389
Methaemoglobin, (MHB, %)	14.52	14.05	15.09	14.48	0.35	0.795
Milk yield (kg/day)	7.10	7.01	7.44	8.01	0.48	0.899
Protein (%)	4.68	4.55	4.75	4.67	0.04	0.348
Fat (%)	8.54^ab^	8.01^a^	9.43^b^	8.95^ab^	0.15	0.008
Total solid content (%)	19.94^ab^	19.31^a^	20.90^b^	20.31^ab^	0.18	0.016
Lactose (%)	5.26^a^	5.38^b^	5.20^a^	5.24^a^	0.02	0.016

Different letters in the same row means significant differences (*P*  < 0.05)

### Fermentation characteristics

The effect of different levels of sodium nitrate on ruminal fermentation parameters is shown in Table 4. Although the effects of nitrate on pH were not significant between control and nitrate treatments (*P * > 0.05), compared to the control group, ruminal pH values were decreased with nitrate supplementation. Here, the concentration of acetate, propionate, butyrate and total VFA in all sodium nitrate-adapted water buffaloes were greater than the control group (*P*  < 0.05, Table 4). However, the ratio of acetate to propionate was not significantly changed (*P*  > 0.05). Compared to the control group, NH_3_–N and MCP concentration was not statistically affected by the addition of sodium nitrate in our study (*P*  > 0.05).

**Table 4:** Effects of different level sodium nitrate on ruminal fermentation parameters

Parameter	Sodium nitrate, g/kg body weight				SE	*P*
	0	0.11	0.22	0.44		
Ruminal pH	6.85	6.66	6.73	6.63	0.04	0.186
Acetate, mM	26.97^a^	35.55^b^	32.67^b^	36.97^b^	1.06	0.001
Propionate, mM	7.02^a^	8.91^b^	8.01^ab^	9.41^b^	0.29	0.010
Butyrate, mM	3.91^a^	5.76^b^	4.57^ab^	5.29^b^	0.20	0.003
A/P	3.93	3.99	4.09	3.98	0.06	0.871
Total VFA, mM	37.91^a^	50.22^b^	45.24^ab^	51.67^b^	1.49	0.001
NH_3_–N mM	22.1^ab^	27.1^b^	13.8^a^	24.3^ab^	1.5	0.022
MCP, mg/ml	0.23	0.29	0.21	0.26	0.01	0.149

Different letters in the same row means significant differences (*P*  < 0.05)

### Milk fatty acids profile

Table 5 shows the effects of sodium nitrate on milk fatty acids profile in water buffaloes. Although the milk fatty acids value at 0.11 g sodium nitrate/kg/d were slightly lower than other treatments, no significant differences were observed among different treatments (*P*  > 0.05). There were no indications that inhibition of methanogenesis by sodium nitrate affected biohydrogenation involving CLA and vaccenic acid (Table 5).

**Table 5:** Effects of sodium nitrate on milk fatty acids profile in water buffaloes

Fatty acids, ppm	Sodium nitrate, g/kg/d				SE	*P*
	0	0.11	0.22	0.44		
C8:0	419.82	344.75	360.24	492.07	27.84	0.229
C10:0	936.66	750.84	851.50	1088.90	60.16	0.238
C11:0	2.87	0.98	3.67	0.99	0.78	0.519
C12:0	1239.70	1023.70	1171.50	1426.90	71.30	0.250
C13:0	41.36	32.06	42.15	40.24	1.95	0.236
C14:0	4874.8^ab^	4563.6^a^	4907.30^ab^	6132.0^b^	213.01	0.045
C14:1 n5	394.40	394.14	388.84	452.03	22.11	0.718
C15:0	497.07	419.49	530.41	520.95	20.79	0.223
C15:1 n5	ND	ND	ND	ND	ND	ND
C16:0	20,676	18,459	21,551	25,071	879.19	0.059
C16:1 n7	833.28	818.57	845.94	981.38	48.23	0.616
C17:0	390.61	347.52	410.51	426.66	15.96	0.331
C17.1 n7	87.10	86.42	84.02	95.35	5.37	0.893
C18:0	7406.1	6919.6	7617.1	8292.7	216.07	0.156
C18:1n9c	8256.2	7653.4	10,715.00	10,195.00	563.64	0.160
C18:1n9t	1389.3	1244.4	1714.2	1448.9	63.74	0.066
C18:2n6c	909.09	837.82	1134.2	1016.9	58.49	0.302
C18:2n6t	ND	ND	ND	ND	ND	ND
C18:1 t11	1837.8	1074.4	1691.4	1522.6	188.17	0.515
C18:2 t9t11	1733.7	1706.5	1790.5	1858.8	129.01	0.978
C18:2 c9c11	656.12	538.28	765.56	802.16	49.19	0.224
C18:2 c9t11	391.41	342.23	450.63	382.53	30.95	0.673
C18:2 t10c12	20.34	0	0	0	5.08	0.397
C18:3n3	ND	ND	ND	ND	ND	ND
C18.3n6	ND	ND	ND	ND	ND	ND
C20:0	137.78	117.79	146.20	148.01	4.85	0.105
C20:1	46.22	47.67	59.23	54.12	3.10	0.423
C20:2	ND	ND	ND	ND	ND	ND
C20:3n3	40.23	34.31	49.43	42.97	2.46	0.180
C20:3n6	ND	ND	ND	ND	ND	ND
C20:4n6	58.67	50.16	71.42	75.84	4.43	0.151
C20:5n3	0.28	1.92	2.08	2.09	0.58	0.638
C21:0	11.03	6.14	8.76	8.74	1.30	0.632
C22:0	39.27	33.17	44.33	41.35	1.66	0.108
C22:1n9	99.65	126.91	118.62	80.10	15.99	0.740
C22:2	ND	ND	ND	ND	ND	ND
C22.6n3	ND	ND	ND	ND	ND	ND
C23:0	10.73	5.67	12.30	10.33	1.70	0.555
C24:0	23.59	19.98	30.16	26.53	1.46	0.089
C24:1	ND	ND	ND	ND	ND	ND
SFA	36,707.41	33,043.24	37,685.61	43,727.34	1447.90	0.068
MUFA	12,954.92	11,452.11	15,625.86	14,837.88	726.39	0.170
PUFA	3809.81	3511.23	4263.75	4584.92	228.12	0.357
UFA/SFA	0.4667	0.4584	0.5136	0.4361	0.0130	0.191

Different letters in the same row means significant differences (*P*  < 0.05)

Sum of SFA reported in this table. Sum of MUFA reported in this table. Sum of PUFA reported in this table. Ratio between the sum of UFA and SFA

### Microbial diversity, microbial population and PCoA analysis

Results of the alpha index of microbial diversity of water buffaloes are resented in Table 6. Good’s coverage values of all groups were higher than 0.99, meaning that most of the bacterial and archaeal 16S rRNA sequences were present in the samples. For bacteria, the richness (ace and chao1) and diversity (Shannon index and Simpson) indices were not significantly different between the control group and nitrate treatments(*P*  > 0.05). For archaea, compared to the control group, the richness (ace and chao1) and diversity (Shannon index) indices were increased by nitrate supplementation (*P*  < 0.05). The Simpson values of archaea were also slightly decreased by sodium nitrate treatments (*P*  > 0.05).

**Table 6:** Effects of sodium nitrate on alpha index of microbial diversity in water buffaloes

	Alpha index	Sodium nitrate g/kg/d				SE	P
		0	0.11	0.22	0.44		
Bacteria	Good’s coverage	0.9916	0.9929	0.9939	0.9908	0.008	0.582
	Chao1	903.45	864.04	902.73	929.36	15.82	0.602
	Shannon index	5.01	5.05	4.94	5.04	0.0625	0.939
	Simpson	0.0231	0.0166	0.0231	0.0280	0.0031	0.698
	Ace	894.84	858.48	894.67	927.81	15.56	0.166
Archaea	Good’s coverage	0.9989	0.9987	0.9986	0.9982	0.0001	0.145
	Chao1	113.03^a^	244.47^b^	208.92^b^	245.08^b^	18.43	0.006
	Shannon index	1.0045^a^	2.2233^b^	2.1112^b^	1.6401^ab^	0.1779	0.027
	Simpson	0.5515	0.3083	0.3052	0.4880	0.0458	0.108
	Ace	136.01^a^	243.57^b^	208.93^b^	245.11^b^	15.79	0.015

Different letters in the same row means significant differences (*P * < 0.05)

Tables 7 and 8 shows the effects of sodium nitrate on the average relative abundance of rumen microbiota (% of total sequences) at the phylum and genus level in water buffaloes. In the archaeal community, *Euryarchaeota* was the only phylum identified. Compared with the control group, sodium nitrate did not affect bacterial abundance at the phylum and genus level, but the relative abundance of the methanogen genera was greatly changed. At the phylum level, the results revealed most of the bacterial sequences belonged to the phyla *Bacteroidetes*, *Firmicutes*, *Proteobacteria*. The percentages of *Bacteroidetes* in control, low, medium and high sodium nitrate treatments were 57.0%, 62.2%, 64.2% and 62.1%, respectively. The percentages of *Firmicutes* in four treatments were 27.9%, 31.3%, 19.4%, 23.1%, respectively. The percentages of *Proteobacteria* in four treatments were 8.6%, 1.4%, 9.1%, 7.6%, respectively. The three phyla accounted for more than 90% of the sequences. Sodium nitrate numerically increased the *Bacteroidetes* phylum. However, there was no statistical difference in this bacterial phylum among the four treatments (*P*  > 0.05).

**Table 7:** Effects of sodium nitrate on average relative abundance of rumen bacterial microbiota (% of total sequences) at the phylum level in water buffaloes (n = 3)

	Phylum	Relative abundance (%)				P value
		0 g/kg	0.11 g/kg	0.22 g/kg	0.44 g/kg	
Bacteria	*Bacteroidetes*	57.040 ± 1.264	62.240 ± 7.488	64.150 ± 12.070	62.070 ± 14.070	0.625
	*Firmicutes*	27.890 ± 7.445	31.320 ± 6.114	19.370 ± 3.265	23.080 ± 23.08	0.184
	*Proteobacteria*	8.628 ± 7.151	1.358 ± 1.071	9.125 ± 7.387	7.592 ± 4.690	0.206
	*Spirochaetae*	1.163 ± 0.788	1.653 ± 0.296	1.611 ± 0.418	1.326 ± 0.537	0.746
	*Lentisphaerae*	1.325 ± 1.453	0.797 ± 0.940	2.040 ± 0.516	1.555 ± 1.000	0.445
	*Cyanobacteria*	1.631 ± 2.164	0.502 ± 0.429	1.618 ± 0.991	1.554 ± 1.080	0.381
	*Saccharibacteria*	1.227 ± 0.451	0.924 ± 0.269	0.976 ± 0.803	1.450 ± 0.091	0.184
	*Tenericutes*	0.465 ± 0.065	0.637 ± 0.221	0.398 ± 0.292	0.721 ± 0.130	0.189
	*Actinobacteria*	0.390 ± 0.077	0.173 ± 0.114	0.422 ± 0.363	0.241 ± 0.062	0.180
	*Fibrobacteres*	0.057 ± 0.073	0.195 ± 0.201	0.040 ± 0.055	0.041 ± 0.036	0.726
	*Elusimicrobia*	0.033 ± 0.028	0.056 ± 0.046	0.061 ± 0.055	0.141 ± 0.092	0.449
	*Unclassified_k__norank*	0.049 ± 0.035	0.076 ± 0.058	0.077 ± 0.059	0.072 ± 0.082	0.888
	*SHA-109*	0.067 ± 0.101	0.041 ± 0.049	0.056 ± 0.052	0.089 ± 0.050	0.774
	*Verrucomicrobia*	0.008 ± 0.003	0.011 ± 0.015	0.022 ± 0.034	0.029 ± 0.017	0.421
	*Synergistetes*	0.006 ± 0.001	0.009 ± 0.008	0.027 ± 0.016	0.026 ± 0.012	0.172
	*Chloroflexi*	0.020 ± 0.014	0.017 ± 0.011	0.010 ± 0.009	0.016 ± 0.007	0.728

**Table 8:** Effects of sodium nitrate on average relative abundance of rumen microbial microbiota (% of total sequences) at the genus level in water buffaloes (n  = 3)

	Genus	Relative abundance (%)				P value
		0 g/kg	0.11 g/kg	0.22 g/kg	0.44 g/kg	
Bacteria	*Prevotella*	27.670 ± 13.190	42.720 ± 6.623	42.650 ± 13.930	33.770 ± 14.870	0.4987
	*Christensenellaceae_R-7*	8.090 ± 3.325	9.894 ± 3.728	5.634 ± 1.645	8.191 ± 7.472	0.4698
	*Bacteroidales_BS11*	8.432 ± 8.314	2.346 ± 1.803	3.342 ± 1.781	13.110 ± 11.580	0.4721
	*Rikenellaceae_RC9*	8.156 ± 4.527	4.384 ± 1.952	6.318 ± 3.029	6.783 ± 1.719	0.5267
	*Acetobacter*	6.639 ± 6.279	0.156 ± 0.130	7.121 ± 6.220	4.391 ± 2.977	0.1743
	*Bacteroidales_RF16*	5.251 ± 4.276	1.602 ± 1.431	4.054 ± 2.045	2.693 ± 1.497	0.4568
	*Ruminococcaceae_NK4A214*	3.461 ± 2.611	5.698 ± 2.056	1.791 ± 0.522	2.219 ± 1.076	0.1811
	*Succiniclasticum*	2.958 ± 2.922	3.227 ± 1.716	1.750 ± 1.044	0.765 ± 0.411	0.2404
	*Prevotellaceae_UCG-001*	2.083 ± 1.298	2.374 ± 1.754	1.595 ± 1.075	0.990 ± 0.842	0.6303
	*Ruminococcus_2*	1.505 ± 0.927	3.530 ± 2.704	0.709 ± 0.555	1.165 ± 0.504	0.4292
	*Bacteroidales_S24-7*	1.989 ± 1.564	3.023 ± 1.262	0.960 ± 0.602	0.724 ± 0.214	0.1816
	*Unclassified_f__Prevotellaceae*	0.766 ± 0.865	3.138 ± 2.861	1.776 ± 1.289	0.742 ± 0.476	0.514
	*Butyrivibrio_2*	2.615 ± 1.908	1.057 ± 0.799	1.291 ± 0.353	1.309 ± 0.834	0.7466
	*Gastranaerophilales*	1.626 ± 2.158	0.498 ± 0.4131	1.612 ± 0.982	1.455 ± 0.953	0.3803
	*Entisphaerae_RFP12*	1.242 ± 1.479	0.681 ± 0.854	1.855 ± 0.477	1.132 ± 0.660	0.3712
	*Treponema_2*	1.004 ± 0.815	1.483 ± 0.366	1.365 ± 0.528	0.953 ± 0.435	0.5641
	*Candidatus_Saccharimonas*	1.202 ± 0.445	0.901 ± 0.267	0.936 ± 0.779	1.408 ± 0.124	0.2109
	*Prevotellaceae_UCG-003*	0.513 ± 0.333	0.638 ± 0.281	0.878 ± 0.421	0.441 ± 0.177	0.542
	*Ruminococcaceae_UCG-014*	0.389 ± 0.230	0.514 ± 0.219	0.525 ± 0.185	0.756 ± 0.277	0.5482
	*Bacteroidales_UCG-001*	0.365 ± 0.144	0.426 ± 0.333	0.751 ± 0.521	0.537 ± 0.143	0.5553
	*Ruminococcaceae_UCG-010*	0.581 ± 0.168	0.445 ± 0.193	0.337 ± 0.089	0.522 ± 0.205	0.3297
	*Saccharofermentans*	0.514 ± 0.239	0.476 ± 0.286	0.374 ± 0.168	0.407 ± 0.197	0.8864
	*Lachnospiraceae_XPB1014*	0.660 ± 0.560	0.249 ± 0.136	0.477 ± 0.164	0.350 ± 0.260	0.4424
	*Lachnospiraceae_NK3A20*	0.440 ± 0.120	0.520 ± 0.116	0.349 ± 0.230	0.388 ± 0.267	0.7265
	*Veillonellaceae_UCG-001*	0.332 ± 0.133	0.274 ± 0.166	0.685 ± 0.512	0.363 ± 0.204	0.7162
	*Ruminiclostridium_5*	0.334 ± 0.396	0.685 ± 0.205	0.231 ± 0.209	0.322 ± 0.224	0.2355
	*[Eubacterium]_coprostanoligenesp*	0.301 ± 0.092	0.164 ± 0.047	0.511 ± 0.479	0.368 ± 0.118	0.1561
	*Erysipelotrichaceae_UCG-004*	0.368 ± 0.278	0.183 ± 0.137	0.270 ± 0.085	0.416 ± 0.090	0.2641
	*Lachnospiraceae_UCG-008*	0.509 ± 0.270	0.216 ± 0.121	0.223 ± 0.150	0.275 ± 0.277	0.5573
	*Succinivibrionaceae_UCG-002*	0.364 ± 0.331	0.476 ± 0.673	0.188 ± 0.058	0.185 ± 0.087	0.7958
Archaea	*Methanobrevibacter*	95.360 ± 4.460	64.690 ± 25.120	69.680 ± 14.650	78.230 ± 8.953	0.091
	Unclassified no rank	1.363 ± 0.464	30.480 ± 24.900	25.470 ± 13.870	16.720 ± 6.179	0.055
	*Thermoplasmatales_Incertae_Sedis*	2.651 ± 3.902	3.289 ± 3.705	4.382 ± 1.699	4.198 ± 2.479	0.917
	*Candidatus_Methanomethylophilus*	0.583 ± 0.999	1.389 ± 0.976	0.212 ± 0.178	0.673 ± 0.651	0.380
	*Methanosphaera*	0.041 ± 0.013	0.104 ± 0.090	0.167 ± 0.115	0.098 ± 0.090	0.368
	*Methanobacteriaceae*	0.003 ± 0.005	0.027 ± 0.038	0.078 ± 0.132	0.053 ± 0.046	0.397
	Unclassified* Euryarchaeota*	0.001 ± 0.003	0.024 ± 0.027	0.014 ± 0.013	0.027 ± 0.021	0.247

Only top 30 were presented

To evaluate the effects of sodium nitrate on the ruminal bacterium community composition, the genera whose abundance were in the top 30 were selected. At the genus level, the results revealed most of the bacterial sequences belonged to the genus *Prevotella, Christensenellaceae_R-7*, *Bacteroidales_BS11*, *Rikenellaceae_RC9*, *Acetobacter*, *Bacteroidales_RF16*, *Ruminococcaceae_NK4A214*, *Succiniclasticum*, and *Prevotellaceae_UCG-001*. The nine genera accounted for more than 70% of the sequences. Five archaeal genera were identified and compared with the control group, about 16.7–30.5% of sequences could not be classified at the genus level (*P*  = 0.055). *Methanobrevibacter* was the most abundant genus and accounted for 95.4% of the total archaeal community in the control group. There was a tendency towards *Methanobrevibacter* to decrease from the sodium nitrate group (*P*  = 0.091).

**Fig. 1:**
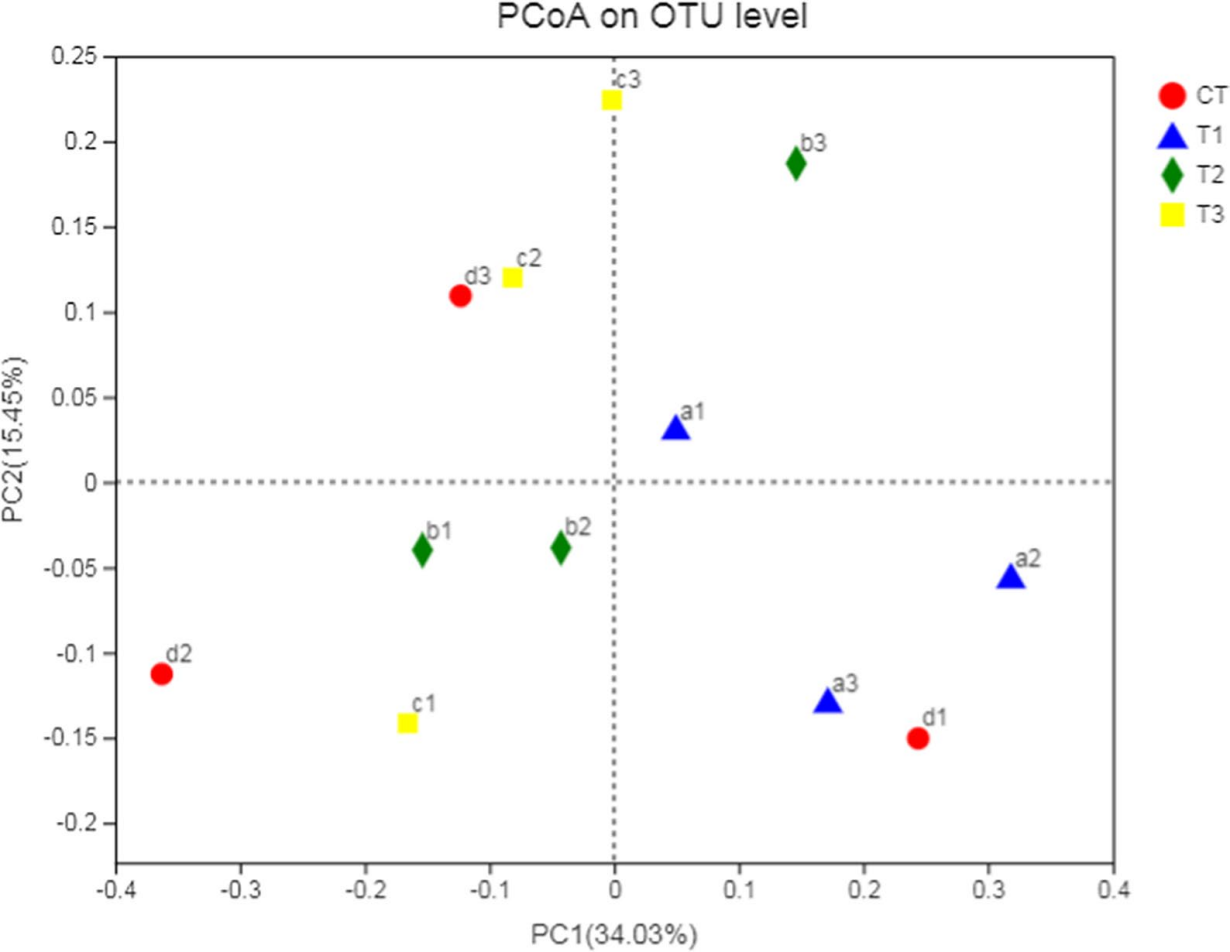
PCoA analysis of bacterial population in the rumen of water buffaloes. CT (a1,a2,a3), T1 (b1,b2,b3), T2 (c1,c2,c3), T3 (d1,d2,d3)

**Fig. 2:**
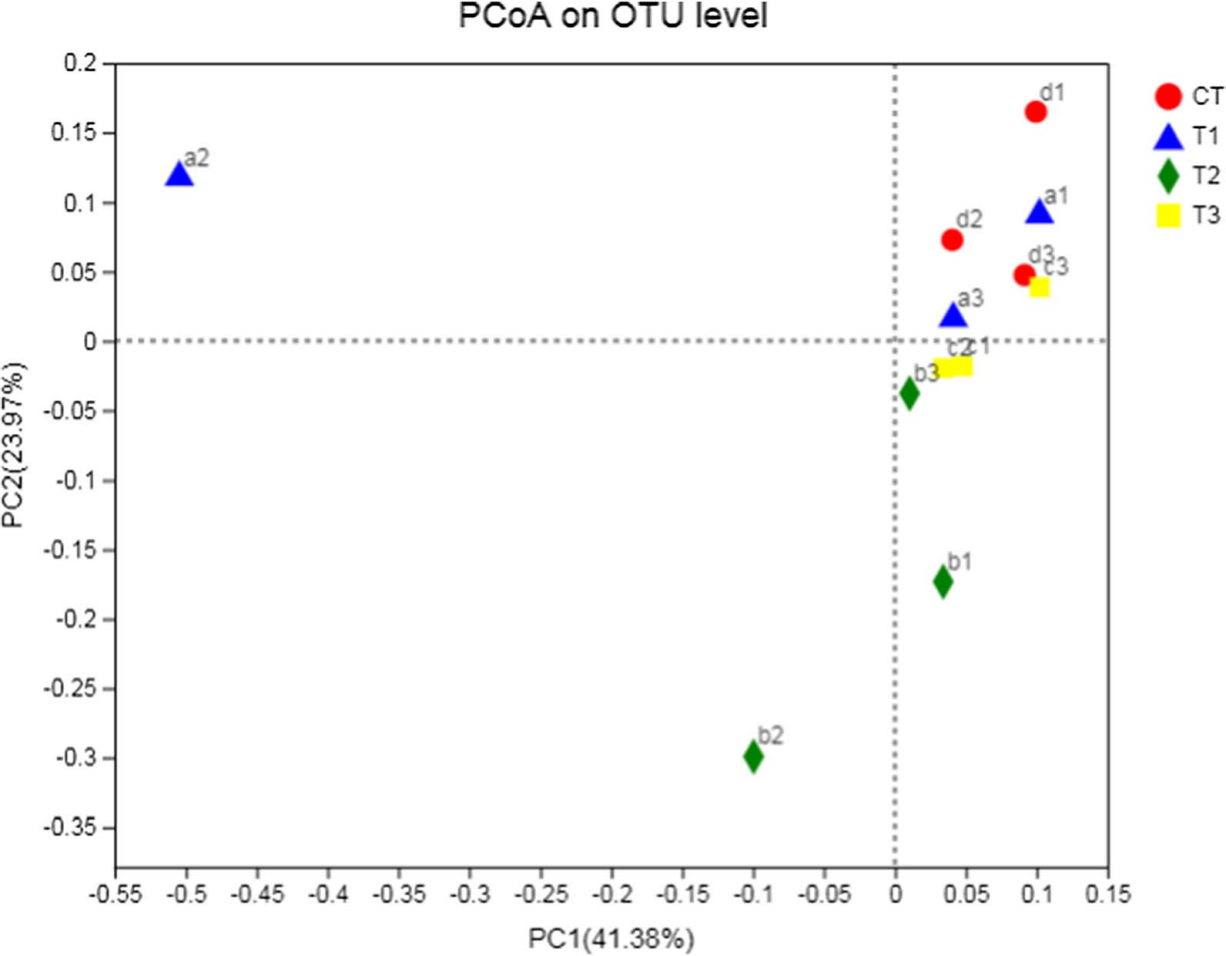
Pocan analysis of archaeal population in the rumen of water buffaloes. CT (a1, a2, a3), T1 (b1, b2, b3), T2 (c1, c2, c3), T3 (d1, d2, d3)

The abundance of total bacteria, archaea, protozoa, fungi and *Butyrivibrio* group species associated with fatty acid biohydrogenation was quantified to assess the impact of sodium nitrate (Table 9). No significant difference in total bacteria, methanogens, fungi, *B. p*r*oteoclasticus, B. fibrsolvens*  +  *Pseudobutyrivibrio *spp., ‘*Atypical*’* Butyrivibrio* and *B. hungatei* abundance was observed between the control group and nitrate treatments (*P*  > 0.05). PCoA analysis of bacterial and archaeal population in the rumen of water buffaloes was presented in Figs. 1 and  2. Comparisons of bacterial communities by principal coordinate analysis (PCoA) based on the Bray–Curtis distance matrix showed no significant separation between the samples obtained from the water buffaloes fed control diet and diets with sodium nitrate (Fig. 1, *P*  = 0.283). However, comparisons of archaeal communities by PCoA analysis showed significant separation between the control group and nitrate treatments (Fig. 2, *P*  = 0.025).

**Table 9:** Effects of sodium nitrate on microbial population in water buffaloes (log_10_ copies/g rumen contents)

Species	Sodium nitrate, g/kg				SE	*P*
	0	0.11	0.22	0.44		
Total bacteria	13.21	13.60	12.46	13.31	0.16	0.108
Protozoa	8.77	9.00	7.51	8.46	0.23	0.142
Methanogenic archaea	8.50	9.05	7.76	8.65	0.19	0.180
Fungi	7.53	7.40	7.26	7.58	0.11	0.817
*B. proteoclasticus*	8.75	8.77	7.92	9.21	0.17	0.087
*B. fibrisolvens* + *Pseudobutyrivibrio* spp.	7.64	7.70	7.05	8.12	0.20	0.410
‘*Atypical*’* Butyrivibrio*	8.43ab	8.67ab	7.68a	9.27b	0.19	0.033
*B. hungatei*	8.05	8.33	7.21	8.40	0.19	0.154

Different letters in the same row means significant differences (*P* < 0.05)

### Correlation analysis

**Fig. 3:**
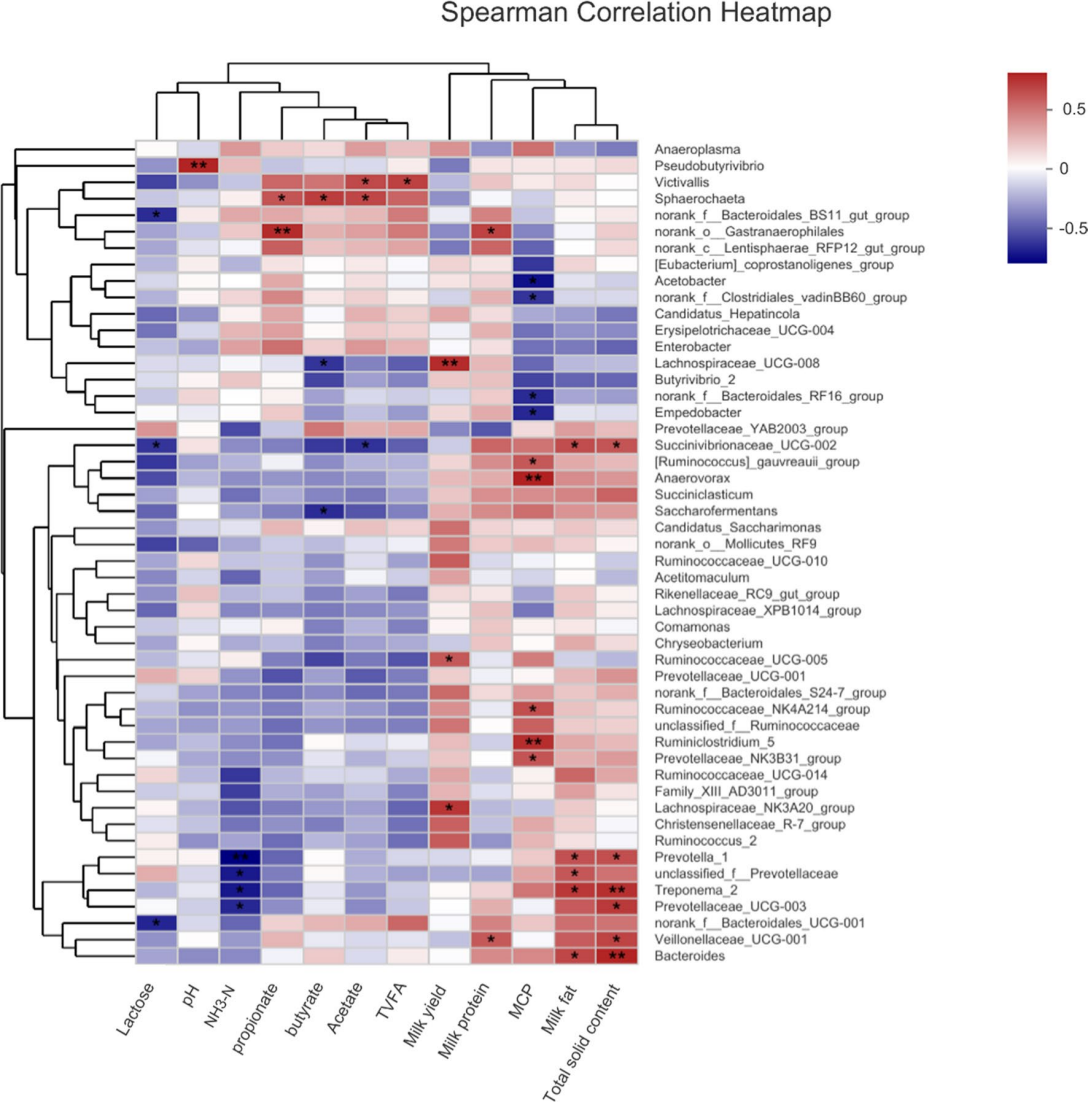
Correlation of bacterial genera with pH, VFA, milk composition, milk yield, NH_3_–N and MCP

The correlation among pH, VFA, milk composition, milk yield, NH_3_–N, MCP and dominant bacterial genera were evaluated (Fig. 3). The results showed that rumen pH correlated positively with *Pseudobutyrivibrio* abundance (r  = 0.759, *P*  = 0.004). Propionate concentration correlated positively with the abundance of *Victivallis* (r  = 0.531, *P*  = 0.075) and *Gastranaerophilales* (r  = 0.734, *P*  = 0.007). Butyrate concentration correlated positively with *Sphaerochaeta* abundance (r  = 0.671, *P*  = 0.016) and negatively with the abundance of *Lachnospiraceae*_UCG-008 (r  = − 0.580, *P*  = 0.048) and *Saccharofermentans* (r  = − 0.606, *P*  = 0.037). Acetate correlated positively with the abundance of *Victivallis* (r  = 0.636, *P*  = 0.026) and *Sphaerochaeta* (r  = 0.643, *P*  = 0.024) and negatively with *Succinivibrionaceae*_UCG-002 abundance (r  = 0.587, *P*  = 0.045). Total VFA concentration correlated positively with *Victivallis* abundance (r  = 0.662, *P*  = 0.019). MCP concentration positively correlation with the abundance of (*Ruminococcus*)_*gauvreauii*_group (r  = 0.580,*P*  = 0.048), *Anaerovorax* (r  = 0.768, *P*  = 0.004), *Ruminococcaceae*_NK4A214_group (r  = 0.615, *P*  = 0.033), *Ruminiclostridium* 5 (r  = 0.727, *P*  = 0.007), *Prevotellaceae*_NK3B31_group (r  = 0.594, *P*  = 0.042) and negatively with the abundance of *Acetobacter *(r  = − 0.683, *P*  = 0.014), norank_f__*Clostridiales_vadin *BB60_group (r  = − 0.578, *P*  = 0.049), norank_f__*Bacteroidales*_RF16_group (r  = − 0.608,*P*  = 0.036) and *Empedobacter* (r  = − 0.618, *P*  = 0.032) genera. Milk yield correlated positively with the abundance of *Lachnospiraceae* UCG-008 (r  = 0.734, *P*  = 0.007), *Ruminococcaceae* UCG-005 (r  = 0.581, *P*  =  0.047) and *Lachnospiraceae*_NK3A20_group (r  = 0.699, *P*  = 0.011). Milk protein correlated positively with the abundance ofnorank_o__*Gastranaerophilales* (r  = 0.655, *P*  = 0.021) and *Veillonellaceae* UCG-001 (r  = 0.581, *P*  = 0.048). The abundance of *Succinivibrionaceae*_UCG-002 (r  = 0.622, *P*  = 0.031; r  = 0.594, *P*  = 0.042), *Prevotella* 1 (r  = 0.608, *P*  = 0.036; r  = 0.615, *P*  = 0.033), *Treponema* 2 (r  = 0.692, *P*  = 0.013; r  = 0.720, *P*  = 0.008) and *Bacteroides* (r  = 0.643, *P*  = 0.024; r  = 0.762, *P*  = 0.004) displayed a positive correlation with milk fat and total solid contents.

**Fig. 4:**
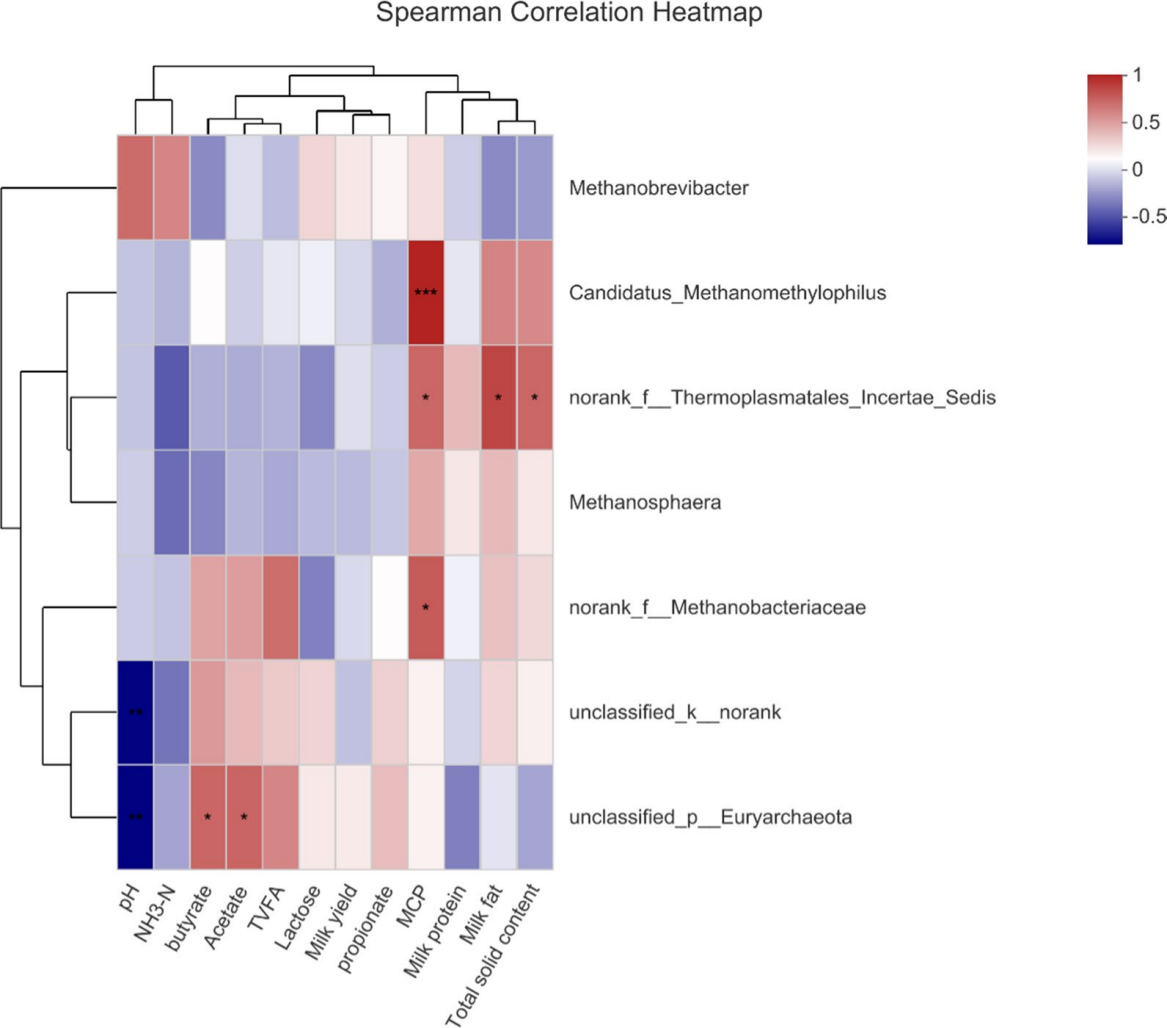
Correlation of archaeal genera with pH, VFA, milk composition, milk yield, NH_3_–N and MCP

The relationships between the milk yield, milk composition, pH, VFA, NH_3_–N, MCP concentration and archaea proportions at the genus levels were evaluated (Fig. 4). The results showed that ruminal pH correlated negatively with the abundance of unclassified_k__norank (r  = − 0.752; *P*  = 0.005) and unclassified_p__*Euryarchaeota* (r  = − 0.759; *P*  = 0.004). Both acetate (r  = − 0.587; *P*  = 0.045) and butyrate (r  = 0.583; *P*  = 0.046) were correlated positively with the abundance of unclassified_p__*Euryarchaeota*. MCP correlated positively with the abundance of *Candidatus_Methanomethylophilus* (r  = 0.825, *P*  = 0.001), norank_f__*Thermoplasmatales_Incertae_Sedis* (r  = 0.580, *P*  = 0.048) and norank_f__*Methanobacteriaceae* (r  = 0.625, *P*  = 0.030). Both milk fat (r  = 0.699, *P*  = 0.011) and total solid contents (r  = 0.580, *P*  = 0.047) were correlated positively with the abundance ofnorank_f__*Thermoplasmatales_Incertae_Sedis*. The other parameters have no significant correlations with archaea genera.

## Discussion

### Blood methaemoglobin, DMI, milk yield and milk composition

Nitrate has been recognized as a useful and promising feed additive to reduce methane emissions from ruminants. In our present study, the nitrate concentrations of the diets and water were not determined because the basal diet and water fed to animals is the same for all treatments. Toxic levels of nitrate in feed are usually associated with excessive levels of dietary crude protein leading to high NH_3_ concentration in the rumen (Leng 2008). At lower inclusion levels, most ingested nitrate can convert to NH_3_–Ninthe rumen, providing nitrogen for the microbial growth (Lewis 1951). Most studies have investigated the potentially toxic effects of nitrates on ruminants; however, it seems that clinical toxicity occur only when animals were given high doses of nitrate salts without a period of adaptation (Eckard 1990). The degree of intoxication can be reflected by the percentage of total haemoglobin in the blood of animals (Leng 2008). Signs of hypoxia develop when 20–30% of haemoglobin is converted to methaemoglobin. Death results when there is 70–80% of methaemoglobin (Vermunt and Visser 1987). Methaemoglobin percentages of this experiment ranged from 10.2 to 17.8%, which means that the water buffaloes in this experiment were not compromised by methaemoglobin. The results are consistent with some previous in vivo studies where feeding nitrate salts did not result in clinical signs of toxicosis (Nolan et al. 2010; Van Zijderveld et al. 2011). However, other studies reported that intoxication is observed in animals when fed nitrate (Takahashi et al. 1998; Sar et al. 2004). The difference in MHB values between our study and these experiments may be caused by the means of application to the animal and nitrate dosages. In the present study, sodium nitrate was fed as a component of the diet, however, in the other studies, the nitrate was pulse-dosed into the rumen. Another possible reason could be the difference in animals that were not adapted to nitrate. Normally, the ruminal microbiota of adapted animals had a higher capacity to reduce nitrite than unadapted animals. Although some strategies (encapsulated nitrate, probiotics (Sar et al. 2005a) can be used to reduce the toxicity of nitrite when nitrate is used to methanogenesis inhibition, more in vivo studies for water buffaloes, including rates of metabolism of nitrate and nitrite, are still needed to understand the necessity for an acclimation strategy in these animals.

The benefits of methane reduction of ruminants (e.g., increasing propionate, butyrate and metabolizable energy supply) have been confirmed (Wolin 1960). When the blood methaemoglobin is higher than 20–30% by feeding nitrate, the ruminant’s productivity will be decreased (Bruning-Fann and Kaneene1993). However, some studies reported that no adverse effects on feed intake and production when nitrate supplementation is at lower levels (Lee and Beauchemin 2014). Here, the nitrate additive treatments did not influence DMI, milk performance or milk composition except that lactose concentrations of milk were higher than control at 0.11 g sodium nitrate/kg body weight (Table 3). Our results agree with previous studies in cattle (Newbold et al. 2014; Olijhoek et al. 2016). In contrast, other studies observed lower DMI when nitrate was fed to animals (Hulshof et al. 2012; Lee et al. 2015; Klop et al. 2016). This difference in the effects of nitrate on DMI may be contributed by the difference in diet compositions, animal species, and feeding strategy. Van Zijderveld et al. (2011) reported that fed nitrate at 2.1% of dietary DM had no adverse effects on milk production and milk composition of dairy cows. The nitrate level should be less than 2.5% of dietary DM, otherwise, the feed intake, as well as animal production, could be affected because of excessive rumen degradable protein from the non-protein nitrogen in the diet (Lee and Beauchemin 2014). Lichtenwalner et al. (1973) found that potassium nitrate of 1.0 or 2.0% in corn gluten meal- and soybean meal-based diets did not affect the growth performance of adapted finishing beef steers. There is no adverse effect on milk production when adequate time was allowed for cows’ adaptation to the nitrate diet, even dietary inclusion of up to 5% of average daily feed intake (Farra and Satter 1971).

### Fermentation characteristics

In the present study, ruminal pH values were decreased with nitrate supplementation, although the changes were minor and not likely to have significant effects on microbial growth or metabolism. This result was in agreement with the previous report that nitrate treatment may lead to a lower ruminal pH (Sar et al. 2004).

In contrast, some studies found that nitrate caused an increase in ruminal pH when using orchard grass forage as the substrate in vitro (Takahashi et al. 1989) or sheep (Nolan et al. 2010). Both type and level of carbohydrate supplementation can contribute to the dynamics of rumen pH (Wanapat et al. 2021).

Nitrate can convert to nitrite and then inhibit ruminal fermentation if ruminants were not adapted (Guo et al. 2009), while this inhibition can be removed after the animal adapted to dietary nitrate (Zhou et al. 2012). In our present study, the concentration of acetate, propionate, butyrate and total VFA in all sodium nitrate–adapted water buffaloes were greater than the control group. This is consistent with what has been found in a previous study that feeding 25 g nitrate/kg DM caused a greater total VFA concentration of sheep compared with sheep receiving an isonitrogenous amount of urea (Nolan et al. 2010). Nitrate did not consistently affect total VFA concentration, but it did shift the VFA profile to higher acetate, lower propionate and lower butyrate molar proportions. Rumen total VFA concentration was not affected by dietary nitrate treatment in beef cattle, while the proportion of acetic acid and acetate/propionate ratio tended to be greater for the nitrate diet (Hulshof et al. 2012). The increase of acetate concentration and the decrease of propionate and butyrate concentration by nitrate supplementation has been confirmed in many previous reports (Farra and Satter 1971; Nakamura 1975; Takahashi et al. 1989; Sar et al. 2004). In contrast, the in vitro study of Zhou et al. (2011), sodium nitrate greatly decreased the production of acetate and propionate. Farra and Satter (1971) also reported that fed a diet containing 20 g nitrate/kg DM decreased concentrations of total VFA in dairy cows. Ruminal VFA concentration is related negatively to ruminal pH, although the relationship appears to be weak because of large variation between diets in removal, buffering and neutralization of acids in the rumen that affects the relationship between pH and VFA (Dijkstra et al. 2012).

Some research reported that an increased ruminal NH_3_–N concentration is associated with nitrate supplementation (Lewis 1951). Nitrate addition can increase ammonia concentrations through respiratory nitrate ammonification in the rumen (Sar et al. 2005a, 2005b) and high ammonia concentrations can inhibit methanogens (Chen et al. 2007, 2008). Increasing ruminal NH_3_–N could provide a ruminal nitrogen source leading in increased microflora and increased microbial protein synthesis and rumen fiber digestion (Khejornsart et al. 2011). In contrast, a difference in NH_3_–N was not expected in the present study. This was consistent with Nolan et al. (2010) which reported no difference in ruminal NH_3_–N between the sheep fed urea or nitrate. Factors which may explain this difference are the different roughage source and type of protein in the diet (true protein and non-protein nitrogen), supply of fermentable energy and efficiency of microbial protein synthesis (Wanapat et al. 2014). MCP concentration was also not statistically affected by the addition of sodium nitrate in our study, which is consistent with the report of Lund et al. (2014). Ruminal MCP synthesis depends mainly on an adequate supply of carbohydrates as an energy source for the synthesis of peptide bonds (Bach et al. 2005).

### Milk fatty acids profile

The effect of feeding nitrate on milk fatty acids profile is little known in water buffaloes or other ruminant species, however, it might be anticipated that the diversion of reducing equivalents away from methanogenesis could have consequences for other processes involving oxidation/reduction reactions such as propionogenesis and fatty acid biohydrogenation. To our knowledge, only one study reported the effect of nitrate on milk fatty acids composition in vivo (Klop et al. 2016). Klop et al. reported that nitrate had no effect on SFA proportion and proportion of MUFA, but increased the proportion of C4:0, C14:0 iso, C15:0 iso, C15:0 anteiso, C16:0 trans-9, C17:0, C18:0, C18:1 trans-10, C18:1 trans-11, C18:2 cis-9, trans-11, C18:3n-6, C20:0 and PUFA in milk fatty acids (Klop et al. 2016). CLA and vaccenic acid (*trans-*11–18:1) are intermediates in the biohydrogenation of linoleic acid to the fully saturated stearic acid (Jenkins et al. 2008). In the present experiment, there were no indications that inhibition of methanogenesis by sodium nitrate affected biohydrogenation involving CLA and vaccenic acid. Our previous in vitro study also suggested that inhibiting methanogenesis have no unintended deleterious consequences on fatty acid metabolism in the rumen (Yang et al. 2019). The reason is probably only about 1–2% of hydrogen was consumed by biohydrogenation as compared to methanogenesis (Nagaraja et al. 1997).

### Microbial abundance changes

*Bacteroidetes* is a major non-cellulosic plant constituent degrader, also an important proteolytic phylum, in the rumen (Thoetkiattikul et al. 2013). *Prevotella* is an important genus of *Bacteroidetes* phylum. In the present study, we observed that the relative abundance of the top genus of *Prevotella* were increased by sodium nitrate. This result is consistent with the previous study (Patra and Yu 2013; Zhao et al. 2015). Low nitrate addition can increase the relative abundance of non-cellulose degraders while high-level nitrate inhibited them (Zhao et al. 2015). In the present study, feeding nitrate caused Chao1, Shannon index and ace values of archaea to be higher than the control group. It indicates the diversion of H_2_ towards nitrate reduction had a significant effect on the archaeal community. The relative abundance of the *Methanobrevibacter* genus in sodium nitrate treatments tended to be lower than the control group whereas the relative abundance of an archaeal genus (unclassified no rank) tended to be increased*.* In contrast, Zhao et al. (2018) found the relative abundance of the majority of the genera in this study, vadinCA11 and *Methanobrevibacter*, was not significantly influenced by nitrate. *Methanosphaera* and *Methanimicrococcus* abundance increased linearly commensurate with increasing nitrate, while *Methanoplanus* abundance was significantly decreased.

*B. proteoclasticus*, *B. fibrsolvens*  +  *Pseudobutyrivibrio* spp., ‘*Atypical*’* Butyrivibrio* and *B. hungatei* are members of *Butyrivibrio* group and carry out the biohydrogenation of unsaturated fatty acids and the formation of CLA and VA (Paillard et al. 2007; Lourenço et al. 2010). In the present study, the population of total bacteria, fungi, methanogens and *Butyrivibrio* group members did not differ from the control group. Our results may indicate that adaptation enabled rumen microorganisms to maintain their abundance in the rumen community. These results were consistent with previous studies. Patra and Yu (2014) similarly investigated the effects of nitrate (5 mM) on the rumen microbial community and abundances of select microbial populations using in vitro methods and found that nitrate treatment did not alter the abundances of total bacteria, *Ruminococcus albus*, or archaea. Zijderveld et al. (2010) reported that the protozoal population in the rumen of lambs was unaffected by the inclusion of nitrate in the diet. Nitrate supplementation did not affect 16S rRNA copies of bacteria, protozoa, methanogens, or fungi (Wang et al. 2018). Lin et al. (2011) found that the nitrate-reducing activity of a fungal fraction from ruminal digesta was low, so their contribution to nitrate metabolism is likely to be minor. In contrast, the populations of representative cellulolytic bacteria, *F. succinogenes*, *R. flavefaciens* and *R. albus*, methanogens, protozoa and fungi were decreased by feeding 9 g/day of potassium nitrate (Asanuma et al. 2015). Compared with the control, archaeal populations were considerably decreased in the nitrate-inoculated media, however, no differences in total bacterial populations (Zhou et al. 2011, 2012). The number of methanogens decreased when nitrate was included in the diet, however, the protozoa population was unaffected (Zijderveld et al. 2010). Many of these differences are likely due to the difference in nitrate dose and perhaps to the different techniques employed for nitrate inclusion. Even after adaptation to dietary nitrate, the relative population sizes for all three putative nitrate-reducing species were very low (Lin et al. 2013). These results indicate that some bacteria can adapt to nitrate or its reduction intermediates, while others probably cannot.

### Association of rumen microbial abundance with fermentation parameters

Correlation analysis indicated a cluster of bacteria positively correlated with pH and VFA, including *Pseudobutyrivibrio*, *Victivallis*, *Gastranaerophilales*, *Sphaerochaeta*, signifying their importance in VFA synthesis. Members of the genus *Pseudobutyrivibrio* are commonly found in the rumen and reported to use a wide range of soluble and some insoluble substrates and characteristically ferment carbohydrates to butyrate, formate, lactate, and acetate (Moon et al. 2008; Kopečnýet al. 2003). *Victivallis* can use fructose as its only energy and carbon source and produces acetate, ethanol, H_2_, and bicarbonate as fermentation products from glucose (Janssen and Hedlund 2011). *Sphaerochaeta* are known to utilize various sugars and to produce volatile fatty acids as fermentation end products. *Gastranaerophilales* belongs to class *Melainabacteria*, which is capable of fermenting arrange of sugars (e.g., glucose, starch and hemicellulose) into butyrate in the gut of herbivores (Rienzi et al. 2013). *Prevotella* is a dominant genus of *Bacteroidetes* phylum and displayed positive correlations with milk fat and total solid contents and negatively with NH_3_–N in this study. *Prevotella* exhibited a lesser importance in methane emissions because it associated with nitrogen metabolism and the pentose phosphate pathway (Hassanet al. 2021; Martínez-Álvaro et al. 2020). In this study, ruminal pH correlated negatively with the abundance of unclassified *Euryarchaeota*. This is agreed with previous study (Diaz et al. 2017). We observed a lower *Euryarchaeota*: *Bacteria* ratio and abundance of *Methanobrevibacter* sp. in the rumen of sodium nitrate supplementation, supported that sodium nitrate is a feed additive that affects enteric methane emissions persistently. This result is consistent with previous study (Granja-Salcedo et al. 2019). The lack of significant correlations between some microbial abundance with VFA does not suggest that those microbial taxa are unimportant. More work needed to explore the correlations, because a small number of species might have a strong impact on rumen fermentation parameters. In conclusion, added 0.11–0.44 g sodium nitrate/kg of body weight increased VFA production and the archaeal richness and diversity indices in water buffaloes but had no effect on milk yield, fatty acids profile, bacterial or archaeal abundance and *Butyrivibrio* group population related to biohydrogenation. Thus, unintended deleterious consequences of lowering methane emissions using dietary nitrate in water buffaloes are unlikely.

## Data Availability

Data and materials will be made available on reasonable request. The raw sequences were deposited in the NCBI Sequence Read Archive (SRA) database (Bacterial Accession Number: SRR11450759; Archaeal Accession Number: SRR11476468).
